# Supplementary material for: Towards fully ab initio simulation of atmospheric aerosol nucleation
Source: Nat Commun. 2022 Oct 14;13:6067. doi: 10.1038/s41467-022-33783-y (PMC9568664; doi:10.1038/s41467-022-33783-y)

Supplementary Information

Towards fully *ab initio* simulation of atmospheric aerosol nucleation

Shuai Jiang, Yi-Rong Liu, Teng Huang, Ya-Juan Feng, Chun-Yu Wang, Zhong-Quan Wang, Bin-Jing Ge, Quan-Sheng Liu, Wei-Ran Guang, Wei Huang

Supplementary Table 1 | Collision rate constants ($cm^3\ s^{-1}$) from deep neutral network force field (DNN-FF)-based molecular dynamics (MD) and the hard sphere model at 278 K and 300 K. The uncertainty of these constants is below 20. k, k_{low} and k_{up} represent the Poisson-based average, the lower and upper bounds of collision rate constants with 95% confidence from DNN-FF-based MD, while k_{gas} represent collision rate constants from the hard sphere collision model.

Temperature (K)	reaction	k ($cm^3\ s^{-1}$)	k_{low} ($cm^3\ s^{-1}$)	k_{up} ($cm^3\ s^{-1}$)	k/k_{low}	k_{up}/k	k_{gas} ($cm^3\ s^{-1}$)	k/k_{gas}	$k_{278\,K}/k_{300\,K}$	reactive events number
278	$(DMA)_1$+$(DMA)_1$->$(DMA)_2$	1.19e-09	6.97e-10	1.88e-09	1.71	1.58	5.68e-10	2.10	2.81	17
	$(SA)_1$+$(SA)_1$->$(SA)_2$	7.92e-10	2.78e-10	1.72e-09	2.84	2.17	3.34e-10	2.37	1.38	4
	$(DMA)_1$+$(SA)_2$->$(SA)_2(DMA)_1$	1.36e-09	4.10e-10	3.20e-09	3.31	2.36	5.26e-10	2.59	0.89	4
	$(DMA)_1$+$(SA)_1$->$(SA)_1(DMA)_1$	1.30e-09	6.90e-10	2.18e-09	1.88	1.68	4.53e-10	2.87	0.74	12
	$(DMA)_1$+$(SA)_1(DMA)_1$->$(SA)_1(DMA)_2$	1.64e-09	6.98e-10	3.20e-09	2.35	1.95	5.67e-10	2.89	1.05	7
	$(DMA)_1$+$(SA)_1(DMA)_2$->$(SA)_1(DMA)_3$	3.88e-09	1.17e-09	9.15e-09	3.31	2.36	6.50e-10	5.97	None	4
	$(DMA)_1$+$(SA)_2(DMA)_1$->$(SA)_2(DMA)_2$	3.84e-09	9.11e-10	1.02e-08	4.21	2.65	6.19e-10	6.21	None	3
	$(SA)_1$+$(SA)_1(DMA)_1$->$(SA)_2(DMA)_1$	2.57e-09	3.90e-10	8.23e-09	6.59	3.20	4.08e-10	6.29	1.13	2
	$(DMA)_1$+$(DMA)_2$->$(DMA)_3$	7.74e-09	2.34e-09	1.83e-08	3.31	2.36	6.28e-10	12.32	None	4
300	$(DMA)_1$+$(DMA)_1$->$(DMA)_2$	4.23e-10	1.49e-10	9.20e-10	2.84	2.17	5.90e-10	0.72	None	6
	$(SA)_1$+$(SA)_1$->$(SA)_2$	5.76e-10	1.74e-10	1.36e-09	3.31	2.36	3.47e-10	1.66	None	4
	$(DMA)_1$+$(SA)_1(DMA)_1$->$(SA)_1(DMA)_2$	1.56e-09	6.62e-10	3.03e-09	2.35	1.95	5.89e-10	2.65	None	7
	$(DMA)_1$+$(SA)_2$->$(SA)_2(DMA)_1$	1.53e-09	2.32e-10	4.89e-09	6.59	3.20	5.46e-10	2.80	None	2
	$(DMA)_1$+$(SA)_1$->$(SA)_1(DMA)_1$	1.75e-09	1.06e-09	2.70e-09	1.65	1.54	4.70e-10	3.72	None	18
	$(SA)_1$+$(SA)_1(DMA)_1$->$(SA)_2(DMA)_1$	2.29e-09	5.45e-10	6.08e-09	4.21	2.65	4.24e-10	5.40	None	3
	$(SA)_1$+$(SA)_2(DMA)_1$->$(SA)_3(DMA)_1$	2.47e-09	3.75e-10	7.90e-09	6.59	3.20	4.47e-10	5.52	None	2
	$(SA)_1$+$(SA)_1(DMA)_2$->$(SA)_2(DMA)_2$	4.36e-09	1.04e-09	1.16e-08	4.21	2.65	4.78e-10	9.11	None	3

Supplementary Table 2 | Metadynamics and active learning sampling structure numbers for the corresponding cluster composition.

Cluster composition	Metadynamics		Active learning
	Metadynamics sampling	DFT calculations	DFT calculations
$(DMA)_1$	99999	69678	2
$(DMA)_2$	50000	15536	22
$(DMA)_3$	100000	4998	2
$(DMA)_4$	0	0	13
$(SA)_1$	257973	58872	2
$(SA)_1(DMA)_1$	120000	25171	84
$(SA)_1(DMA)_2$	50000	10532	447
$(SA)_1(DMA)_3$	0	0	54
$(SA)_1(DMA)_4$	0	0	57
$(SA)_2$	144320	4610	51
$(SA)_2(DMA)_1$	49999	16377	63
$(SA)_2(DMA)_2$	50000	4339	106
$(SA)_2(DMA)_3$	50000	4998	13
$(SA)_2(DMA)_4$	0	0	201
$(SA)_2(DMA)_5$	0	0	14
$(SA)_3$	50303	5028	24
$(SA)_3(DMA)_1$	0	0	3
$(SA)_3(DMA)_2$	49863	4801	62
$(SA)_3(DMA)_3$	49998	4992	51
$(SA)_3(DMA)_4$	49997	4987	12
$(SA)_4$	50000	4014	0
$(SA)_4(DMA)_3$	49997	4991	146
$(SA)_4(DMA)_4$	49867	2315	0
$(SA)_4(DMA)_5$	50293	200	17
$(SA)_5(DMA)_4$	49999	200	0
$(SA)_5(DMA)_5$	50000	200	109
$(SA)_5(DMA)_6$	49999	191	0
Total	1522607	247030	1555
Percentage of total DFT structure number	Null	99.37%	0.63%

Supplementary Table 3 | Molecular dynamics parameters in active learning iterations.

Iteration number	Initial molecular composition[a]	box length	time length (ps)	structures number[b]
0	(4, 4)	40	2	13
1			2	27
2			10	36
3			10	103
4			20	89
5			50	172
6	(5, 5)	50	100	168
7			200	480
8			500	467

Notes: a. (m, n) represents the number of SA and DMA molecules in the cluster, respectively. b. It gives the total cluster structure number derived from each active learning iteration.

Supplementary Figure 1 | Dimer detachment curves for $(SA)_1(DMA)_1$, $(SA)_2$ and $(DMA)_2$, where SA and DMA represent sulfuric acid and dimethylamine molecules, respectively. The relative energy is the isomer energy minus the energy of the most stable isomer. DeePMD represents the model trained by DeePMD with a cut-off radius of 6.0 Å. Source data are provided as a Source Data file.

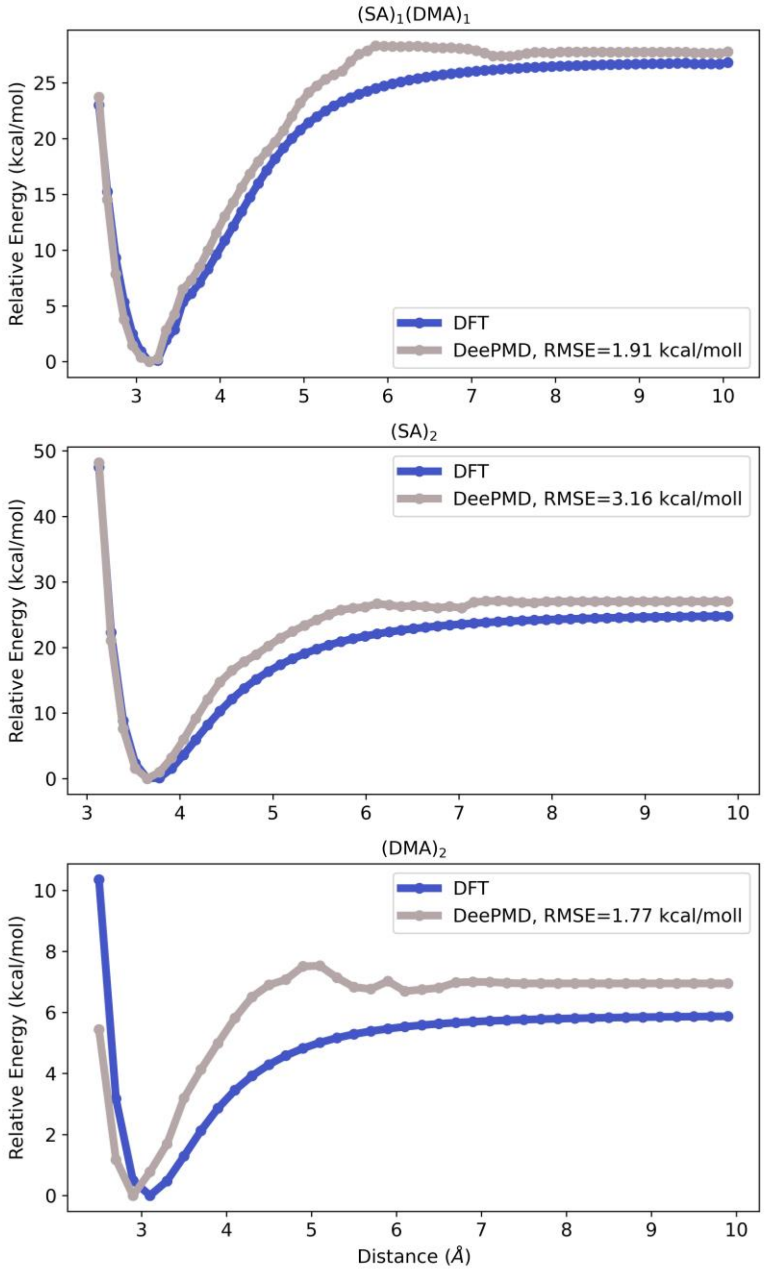

Supplementary Figure 2 | Time dependences of the total energy (the relative value to the first snapshot) and temperature during the MD simulation under NVE ensemble. Source data are provided as a Source Data file.

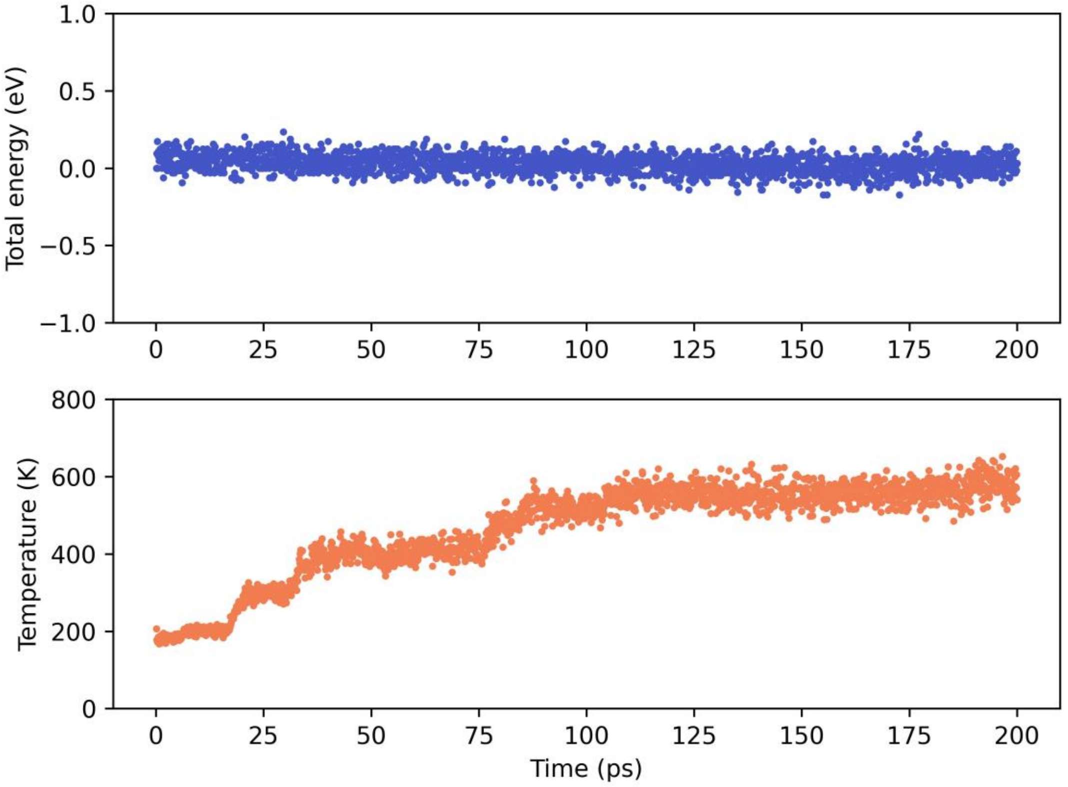

Supplementary Figure 3 | Formation of $(SA)_6(DMA)_6$ in DNN-MD from the collision of $(SA)_2(DMA)_1$ and $(SA)_4(DMA)_5$. Source data are provided as a Source Data file.

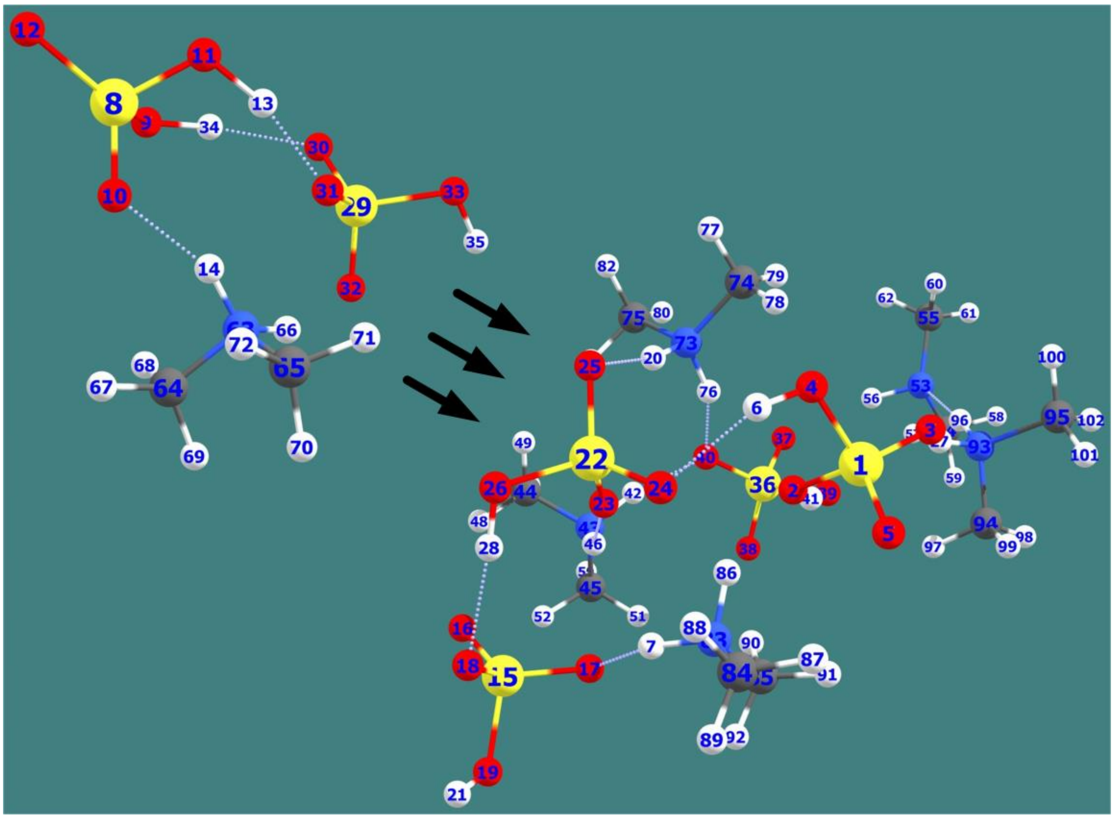

Supplementary Figure 4 | Time evolution of the distance for seven hydrogen oxygen (H-O) atomic pairs in the $(SA)_6(DMA)_6$ cluster. The seven H-O pairs in sulfuric acid molecules are covalently bonded at the very beginning of $(SA)_6(DMA)_6$ formation. Source data are provided as a Source Data file.

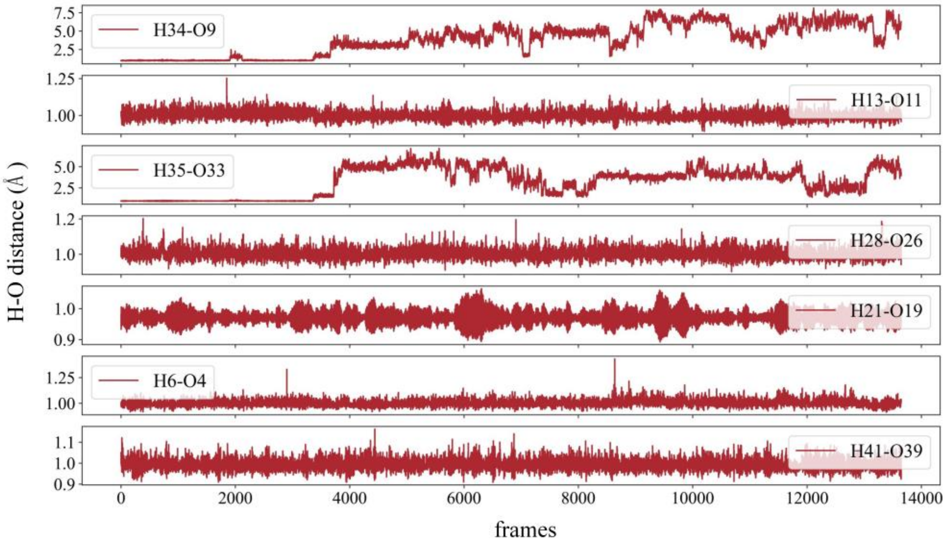

Supplementary Figure 5 | Time evolution of distance for eleven hydrogen nitrogen (H-N) atomic pairs in the $(SA)_6(DMA)_6$ cluster. The eleven H-N pairs are covalently bonded at the very beginning moment of $(SA)_6(DMA)_6$ formation. Source data are provided as a Source Data file.

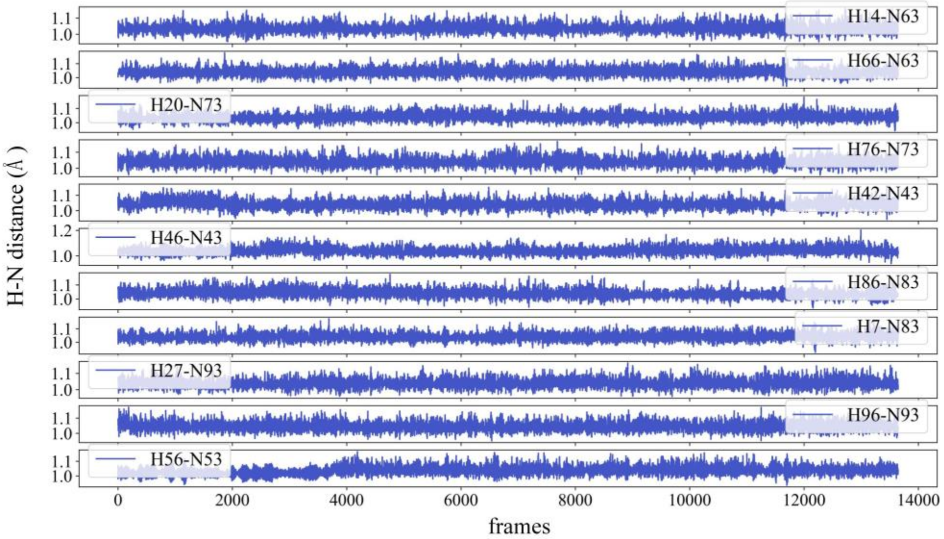

Supplementary Figure 6 | Time evolution of proton number within each sulfuric acid molecule for the $(SA)_6(DMA)_6$ cluster. Notably, the time evolution is filtered with a proton number duration of more than 100 fs to observe the relatively long existence of bonds. Source data are provided as a Source Data file.

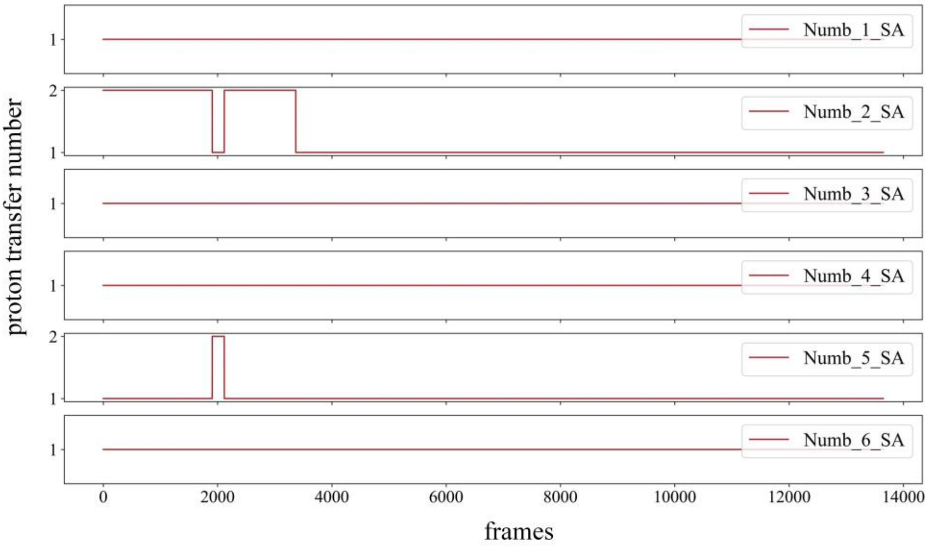

Supplement: Supplementary file 1 — Supplementary Information [file 41467_2022_33783_MOESM1_ESM.pdf]
